# Development of a Novel Intraperitoneal Icodextrin/Dextrose Solution for Enhanced Sodium Removal

**DOI:** 10.1016/j.xkme.2024.100938

**Published:** 2024-11-16

**Authors:** Jennifer L. Asher, Juan B. Ivey-Miranda, Christopher Maulion, Zachary L. Cox, Julian A. Borges-Vela, Genaro H. Mendoza-Zavala, Jose A. Cigarroa-Lopez, Rogelio I. Silva-Rueda, Cristina Revilla-Monsalve, Julieta Moreno-Villagomez, Daniela Ramos-Mastache, Oliver Goedje, Ian Crosbie, Christopher McIntyre, Fredrick Finkelstein, Jeffrey M. Turner, Jeffrey M. Testani, Veena S. Rao

**Affiliations:** 1 Department of Comparative Medicine, Yale University School of Medicine, New Haven, CT; 2 Department of Internal Medicine, Section of Cardiovascular Medicine, Yale University School of Medicine, New Haven, CT; 3 Division of Nephrology, Department of Medicine, Yale University School of Medicine, New Haven, CT; 4 Hospital de Cardiologia, Instituto Mexicano del Seguro Social, Mexico City, Mexico; 5 Hospital de Especialidades, Instituto Mexicano del Seguro Social, Mexico City, Mexico; 6 Department of Pharmacy Practice, Lipscomb University College of Pharmacy, Nashville, TN; 7 Facultad de Estudios Superiores Iztacala, Universidad Nacional Autonoma de Mexico, Mexico City, Mexico; 8 Sequana Medical NV, Ghent, Belgium, London, Canada; 9 Division of Nephrology, Department of Medicine, Schulich School of Medicine and Dentistry, Western University, London, Canada

**Keywords:** Heart failure, icodextrin, peritoneal dialysis, sodium excretion

## Abstract

**Rationale & Objective:**

Peritoneal dialysis (PD) solutions provide both clearance of uremic toxins and sodium and water. An intraperitoneal (IP) solution of icodextrin and glucose designed without the requirement for uremic toxin clearance could provide substantially greater sodium and water removal than PD solutions.

**Study Design:**

We examined varying concentrations of icodextrin and dextrose IP solutions in rats. We evaluated a 30% icodextrin and 10% dextrose IP solution in animals and humans.

**Participants:**

Small and large animal models, and humans (N = 10) with kidney failure.

**Exposure:**

30% icodextrin and 10% dextrose IP solution.

**Outcomes:**

We evaluated ultrafiltration (UF), sodium removal, and peritoneal health in animals. We evaluated safety, tolerability, and efficacy in humans.

**Results:**

In rats, increasing concentrations of icodextrin and dextrose IP solutions, up to 30% icodextrin and 10% dextrose, produced progressively greater UF (*P* < 0.001). In sheep treated with 30% icodextrin and 10% dextrose, the mean UF was ∼3.5-fold greater (1.77 ± 0.22 L vs 0.47 ± 0.34 L; *P* = 0.005) and the mean sodium removal was ∼4-fold greater (7.07 ± 0.72 g vs 1.78 ± 1.27 g; *P* = 0.003) compared with commercially available 7.5% icodextrin PD solution. Long-term exposure of mice (30 days) and sheep (30-45 days) to a 30% icodextrin and 10% dextrose IP solution resulted in no significant structural tissue changes compared with the control 4.25% commercially available PD solution. In humans, a 24-hour dwell of a 30% icodextrin and 10% dextrose IP solution resulted in median net UF of 2,498 mL (IQR, 2,249-2,768), and median sodium removal of 387 mmol (IQR, 372-434 mmol). No serious adverse events occurred.

**Limitations:**

The long-term safety with chronic therapy and the efficacy in patients without kidney failure were not established and require future studies.

**Conclusions:**

A 30% icodextrin and 10% dextrose IP solution provides more efficient UF and sodium removal than traditional PD solutions. The promising inhuman safety and efficacy results warrant future investigation as a sodium removal therapy in patients with edematous disorders such as heart failure.

**Clinical Trial Registration:**

NCT05780086.

**Summary:**

We aimed to design a novel intraperitoneal solution designed for optimal sodium and water removal. A sodium-free 30% icodextrin and 10% dextrose intraperitoneal solution was evaluated in animal models and humans to determine the safety and efficacy. A 30% icodextrin and 10% dextrose solution provides more efficient sodium and water removal than traditional peritoneal dialysis solutions. The promising inhuman safety and efficacy results warrant future investigation as a sodium removal therapy in patients with edematous disorders such as heart failure.

## Plain-Language Summary

We aimed to design a novel intraperitoneal solution designed for optimal sodium and water removal. A sodium-free 30% icodextrin and 10% dextrose intraperitoneal solution was evaluated in animal models and humans to determine the safety and efficacy. A 30% icodextrin and 10% dextrose solution provides more efficient sodium and water removal than traditional peritoneal dialysis solutions. The promising inhuman safety and efficacy results warrant future investigation as a sodium removal therapy in patients with edematous disorders such as heart failure.

Intraperitoneal (IP) solutions for peritoneal dialysis (PD) in kidney failure are primarily designed to optimize clearance of uremic toxins, which limits the amount of sodium and water that can be removed per unit of IP solution.^1^^,^^2^ However, in heart failure (HF) and other edematous disorders, renal toxin clearance is adequate in most patients while renal sodium and water excretion is the primary aberration.^3^^,^^4^ As such, IP solutions can be designed with the principal goal of efficient salt and water clearance for application to these disease states.^5^^,^^6^

We previously provided a proof of concept that a sodium-free, 10% dextrose IP solution increased sodium removal 4-fold compared with an equal volume of a standard 4.25% dextrose commercially available PD solution.^7^ Building upon these promising observations, we sought to develop a dedicated IP solution that satisfies the following conditions: (1) that was designed for optimal sodium and water removal; (2) that was concentrated in a small volume; and (3) that produced overall peritoneal safety superior to purely dextrose-based IP solutions.^5^^,^^8^ The goal was to create a biphasic solution that leveraged the safety and slow-sustained ultrafiltration (UF) of icodextrin for its primary effect with the rapid UF of dextrose. With this approach, a low-volume solution of highly concentrated icodextrin could rapidly “bloom” using dextrose-based UF into a larger volume of moderately concentrated icodextrin (before significant lymphatic uptake of the concentrated solution), thus providing slow continuous UF from the icodextrin component.

In this study, we present the findings from a series of animal experiments and an inhuman trial with the objective of developing and evaluating a sodium-free combined icodextrin and dextrose IP solution for sodium and water removal.

## Methods

The primary aim of the experiments in rats was to evaluate varying concentrations of icodextrin and dextrose sodium-free IP solutions for sodium and water removal. Experiments in pigs and sheep aimed to describe fluid volume and sodium removal kinetics. Experiments in mice and sheep aimed to describe the effect of chronic exposure to icodextrin on peritoneal health. The inhuman feasibility study aimed to measure the direct sodium removal and safety of a novel 30% icodextrin and 10% dextrose IP solution. For studies involving humans and chronic exposure in animals, the 30% icodextrin and 10% dextrose IP solution (DSR Infusate 2.0) was manufactured by Infomed Fluids S.r.l. in compliance with international standards for chemistry, manufacturing, and controls product development.

All animal studies were conducted with approval of the Yale University Institutional Animal Care and Use Committee and according to regulations outlined in the United States Department of Agriculture Animal Welfare Act (9 Code of Federal Regulations, parts 1, 2, and 3) and the conditions specified in the Guide for the Care and Use of Laboratory Animals, Eighth Edition (ILAR publication, 2011, National Academy Press).^9^ The interventional pilot study in humans was approved by the local institutional review board and registered at clinicaltrials.gov (NCT05780086). All patients provided written informed consent before any study procedures were performed.

### Experiments in Rats

Complete methods are provided in Item S1. Briefly, the primary outcome was the change in IP volume. Naive Sprague Dawley rats (*Rattus norvegicus*) (N = 114) were injected IP with 10 mL of a water-based test solution containing combinations of icodextrin (7.5%, 15%, 25%, and 30%) and dextrose (no dextrose, 5%, or 10%). The control group included 24 rats receiving 10% dextrose.

### Experiments in Sheep and Pigs

Complete methods are provided in Item S1. Briefly, the primary outcome was fluid volume and sodium removal kinetics. Six sheep (n = 3 in each group) were exposed to the following 2 different IP solutions: 30% icodextrin and 10% dextrose or 7.5% standard icodextrin infusate with a dwell time of 8 hours. IP fluid was sampled serially. The sheep were euthanized at the end of an 8-hour dwell time, and the total fluid volume was measured. Identical procedures were performed in 6 pigs.

### Good Laboratory Practice Experiments on Chronic IP Health

To evaluate the effects of chronic exposure to a 30% icodextrin and 10% dextrose IP solution on the peritoneum, kidneys, omentum, and peritoneal cavity, experiments in mice and sheep were conducted. Complete methods are provided in Item S1.

### Pilot Study in Humans

We conducted an open-label, single-arm, single-center, study of short-term safety, tolerability, and efficacy of a sodium-free 30% icodextrin and 10% dextrose IP solution in 10 patients with kidney failure receiving chronic PD (Chihuahua2022; NCT05780086). Full inclusion and exclusion criteria and a study diagram (Fig S1) are described in Item S2. Key inclusion criteria were an age of 18 years or older, kidney failure receiving PD with a PD prescription unchanged in the previous month, and an euvolemic volume status per the treating physician. Key exclusion criteria included serum sodium < 130 mEq/L, serum bicarbonate < 18 mEq/L, or active or suspected peritonitis. A volume of 500-mL 30% icodextrin and 10% dextrose IP solution was infused into the peritoneal cavity via a PD catheter with a 24-hour dwell time.

Safety was assessed by counting serious adverse events related to the infusate, monitoring of vital signs (blood pressure, heart rate, respiration rate, oxygen saturation, and body temperature), and blood analyses (eg, urea, creatinine, glucose, sodium, potassium, magnesium, calcium, phosphorus, bicarbonate, and albumin) during the dwell time (baseline, 3, 6, 12, 18, and 24 hours).

Tolerability was assessed using a patient questionnaire measuring pain on a numerical scale of 0-4 (at infusion start; at 1, 3, and 5 minutes from infusion start; at the end of infusion; 15 and 30 minutes after infusion end; every hour after infusion end thereafter, until end of dwell time and at the start of drain; and 5 minutes later and at the end of the drain) and by application of the McGill pain questionnaire at 30 minutes after the end of the infusion. The treatment was considered tolerable if a participant completed the 24-hour dwell time without reporting grade 3-4 pain that led to discontinuation of the dwell.

Efficacy was evaluated as the UF volume and the total sodium excretion assessed by the following aspects: (1) the total UF volume at the end of the 24-hour dwell time and (2) the total sodium excretion at the end of the 24-hour dwell time (concentration of sodium in the IP fluid sample at the end of dwell time multiplied by the total drained volume). Sodium removal was monitored by measuring the sodium concentration in the PD liquid at every 15 minutes to every hour during dwell time. Any change in plasma glucose concentration was assessed by blood analysis. All patients provided informed consent before study procedures.

### Assays and Calculations

Sodium, glucose, and icodextrin concentrations in IP fluid were determined using a Roche fully automated chemistry autoanalyzer (Roche Diagnostics). I-131-albumin concentration in IP fluid was determined using gamma counting using either a Cobra Gamma Counter (Canberra-Packard Corp) or a Daxor BVA device (Daxor Inc). Peritoneal volumes were calculated based on count reduction and the known instilled volume. The absolute quantity of IP solute was calculated using the I-131 albumin–derived volume multiplied by the solute concentration in the peritoneal fluid. Net UF was calculated by subtracting the volume instilled from the total drained volume.

### Statistical Analysis

Continuous data are shown as the mean ± standard deviation or median (quartile 1 to quartile 3) according to the observed distribution. Categorical data are shown as frequency (percentage). For experiments in rats, *t* test was used to assess the effect of icodextrin on IP fluid mass. Linear regression was used to estimate the effects of water, 5% dextrose, or 10% dextrose on IP fluid mass, as well as the linear trend of icodextrin concentration (7.5%, 15%, 20%, 25%, and 30%) in each of these groups. For experiments in pigs and sheep, *t* test was used to assess the effect of icodextrin on the total UF and the total sodium removed. For Good Laboratory Practice experiments (mouse or sheep), *t* test was used to compare continuous variables between groups. Statistical significance was defined as a 2-tailed *P* value of <0.05. Statistical analyses were performed with IBM SPSS Statistics version 26 (IBM Corp) and Stata SE version 16.0 (StataCorp).

## Results

### Effect of Icodextrin on UF and Sodium Removal in Animals

#### Dose-Ranging Experiment in Rats

In rats, increasing concentrations of icodextrin and dextrose IP solutions resulted in progressively greater UF (*P* < 0.001). The greatest UF volume was observed with a 30% icodextrin and 10% dextrose IP solution (Fig S2). The combination of icodextrin and dextrose was additive with 30% icodextrin and 10% dextrose producing 36% more UF than 30% icodextrin in water (*P* = 0.006).

#### Large Animal Evaluation

Total UF was more than 3.5 times greater in sheep infused with 30% icodextrin and 10% dextrose compared with sheep infused with a standard 7.5% icodextrin dialysate solution (mean 1.77 ± 0.22 L vs 0.47 ± 0.34 L; *P* = 0.005; Fig S3). Total sodium removed was approximately 4 times greater with 30% icodextrin and 10% dextrose compared with a standard 7.5% icodextrin dialysis solution (7.07 ± 0.72 g vs 1.78 ± 1.27 g; *P* = 0.003). (Item S3) There was significant IP icodextrin metabolism in the pig models but not in sheep (Figs S3-S5), indicating that swine are not an ideal model to study icodextrin kinetics (Item S4).

#### Chronic Peritoneal Effects of Icodextrin

In mice and sheep chronically exposed to a 30% icodextrin and 10% dextrose IP solution, no significant differences in the gross appearance or histopathology of peritoneum, kidneys, omentum, or peritoneal cavity were observed between icodextrin and control groups. Full results are available in Item S5.

### Phase 1 Study in Humans

Baseline characteristics of the patients (n = 10) are provided in Table 1. All patients completed the 24-hour dwell time with a 30% icodextrin and 10% dextrose IP solution. The median 24-hour urine output during the study visit was 401 mL (interquartile range [IQR], 49-711). No patients died or experienced a serious adverse event. In total, 22 adverse events occurred (Table 2), of which 18 (81.8%) were deemed at least possibly related to study treatment. Three patients with severe hypertension at baseline had a systolic blood pressure decrease of 40 mm Hg or more, but no patients had systolic blood pressure less than 100 mm Hg or symptoms of hypotension. Overall, the test solution was well tolerated by the participants, with only 1 patient reporting significant pain (Fig S6). This occurred at the end of IP instillation of the test solution and resolved within 15 minutes post infusion.

**Table 1:** Phase 1 Trial Baseline Characteristics

Characteristic	Patients (N = 10)
Age (y)	
Median (IQR)	58 (51-69)
Biologic sex, n (%)	
Male	7 (70)
Female	3 (30)
Medical history, n (%)	
Type 2 diabetes	6 (60)
Hypertension	10 (100)
Hyperlipidemia	7 (70)
Abdominal surgery	4 (40)
Heart failure	0 (0)
Kidney transplant	0 (0)
Social history, n (%)	
Tobacco use	4 (40)
Alcohol or illicit drug use	0 (0)
Kidney failure	
Etiology of kidney failure, n (%)	
Type 2 diabetes	6 (60)
Hypertension	0 (0)
Glomerulonephritis	0 (0)
Idiopathic	3 (30)
Other (nephrolithiasis)	1 (10)
Duration of PD requirement (y)	
Median (IQR)	2 (1,4)
Type of PD, n (%)	
CAPD	1 (10)
APD	9 (90)
Urine output > 100 mL/24 h	7 (70)
24-H urine output (mL)	401 (49-711)

*Note*: data are presented as N (%) and median (IQR) as indicated.

Abbreviations: APD, ambulatory peritoneal dialysis; CAPD, continuous ambulatory peritoneal dialysis; IQR, interquartile range; PD, peritoneal dialysis.

**Table 2:** Phase 1 Trial Adverse Events

Adverse Events, N (%)	Patients (N = 10)
Death	0 (0)
Serious adverse events	0 (0)
Nonserious adverse events	
Abdominal pain	8 (80)
Cramps	3 (30)
Nausea	1 (10)
Vomiting	2 (20)
Gastric fullness	1 (10)
Diaphoresis	1 (10)
Decrease in SBP > 40 mm Hg without hypotension^a^	3 (30)
SBP < 100 mm Hg	0 (0)
Symptomatic hypotension	0 (0)
Anxiety	1 (10)
Pruritus	1 (10)
Chills	1 (10)
Discontinuation of therapy due to adverse events	
Serious adverse event	0 (0)
Nonserious adverse event	0 (0)

*Note:* data are presented as N (%) during the 24-h dwell time.

Abbreviation: SBP, systolic blood pressure.

a In these 3 patients, the average SBP at baseline was 186 mm Hg (>175 mm Hg in all), and the SBP decreased to average SBP of 117 mm Hg (≥110 mm Hg in all). No patients had symptoms of hypotension.

**Figure 1:**
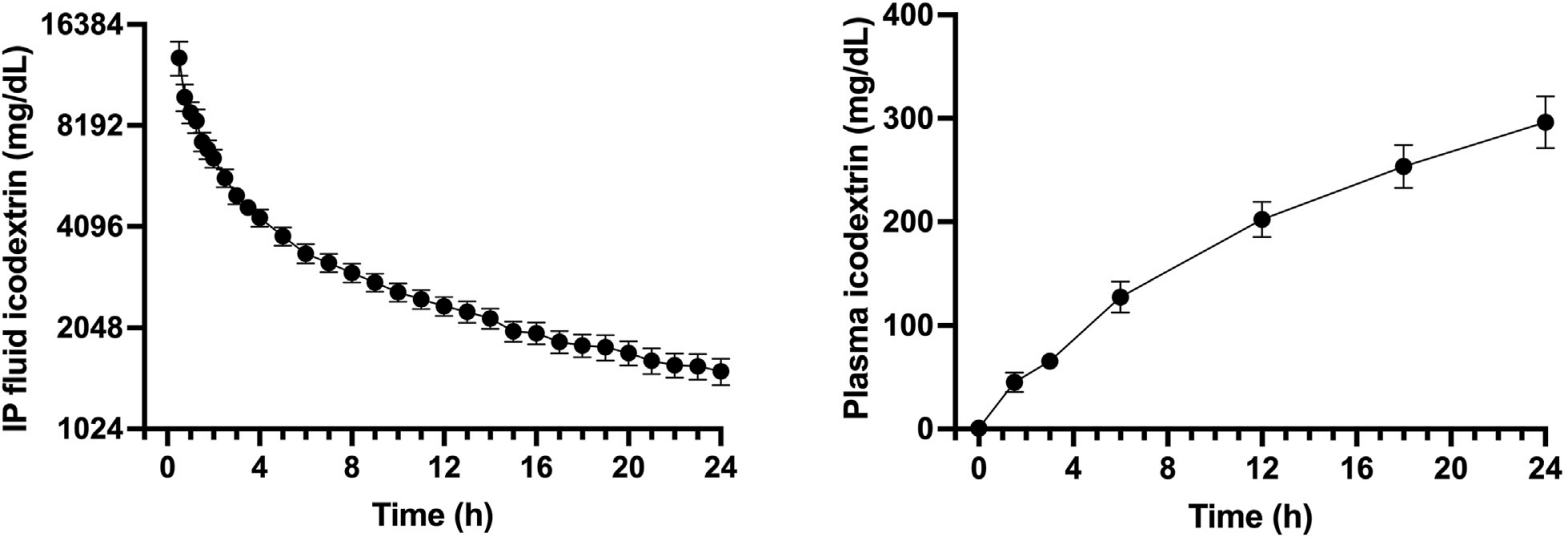
Kinetics of intraperitoneal (IP) and plasma icodextrin. Humans (N = 10) underwent a 24-hour dwell with a 30% icodextrin and 10% dextrose IP solution. The IP (left) and plasma (right) icodextrin concentrations over 24 hours are shown as mean (standard error of the mean).

**Figure 2:**
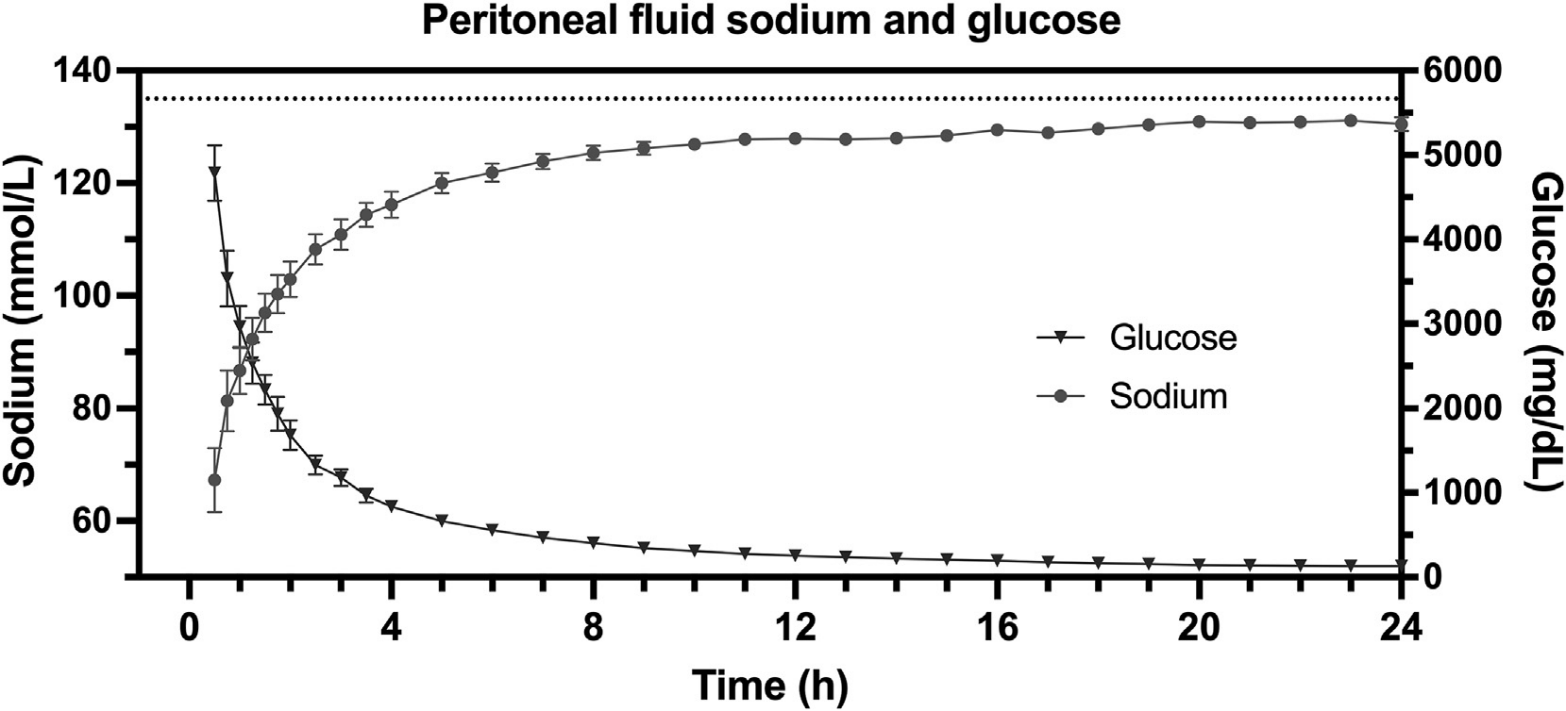
Sodium and glucose concentrations in the peritoneal fluid over time. The intraperitoneal fluid sodium (circles) and glucose (triangles) concentrations are graphed over 24 hours as mean (standard error of the mean) with the horizontal dotted line representing the serum sodium concentration over the same time period.

**Figure 3:**
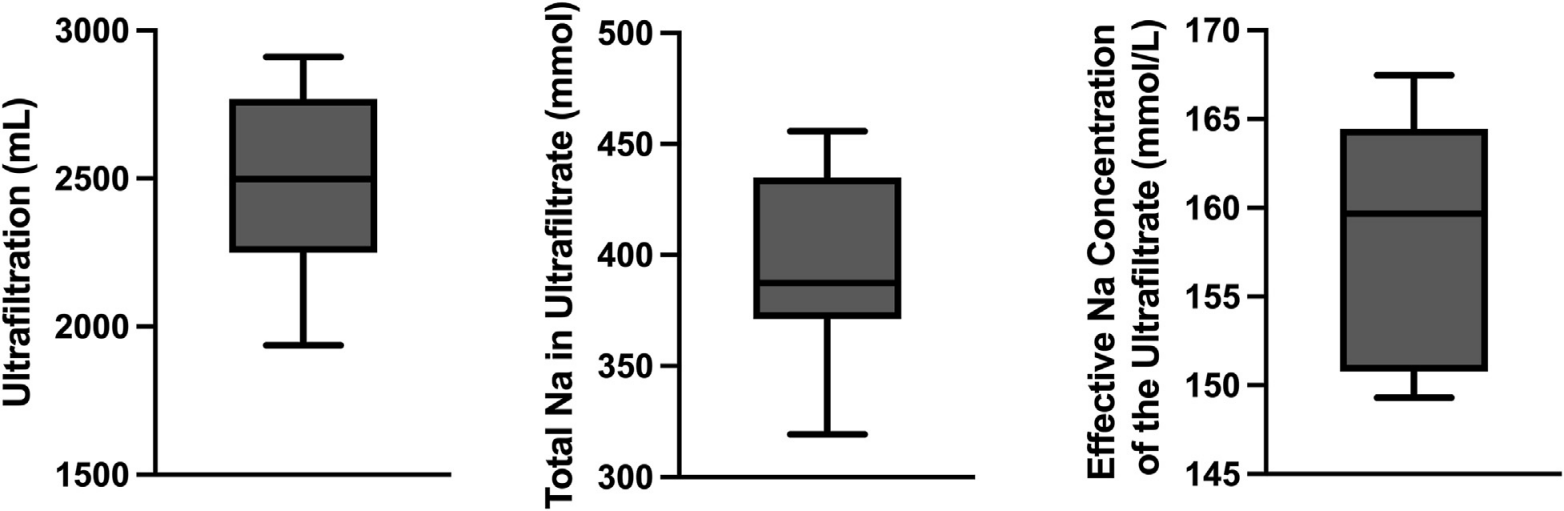
Ultrafiltration and sodium removal. The net ultrafiltration volume (left), total sodium (mmol) in the ultrafiltrate (middle), and effective sodium concentration of the ultrafiltrate drained at the end of the 24-hour treatment period (right) are shown as median (interquartile range). Net ultrafiltration is calculated as the volume of the intraperitoneal fluid drained at 24 hours after subtracting the 500 mL of the 30% icodextrin and 10% dextrose intraperitoneal solution instilled. Effective sodium concentration of the ultrafiltrate is calculated as the total sodium in the ultrafiltrate (mmol) divided by the net ultrafiltration (mL).

The icodextrin IP concentration decreased over the 24-hour study period as the UF volume increased (Fig 1). Plasma levels of icodextrin and nonglucose icodextrin metabolites increased over the 24-hour dwell time. The IP fluid sodium concentration increased rapidly over the first ∼8 hours with a slower rate of increase over the remainder of the 24-hour period (Fig 2). The median total volume drained from the peritoneal cavity after a 24-hour dwell time was 3,031.5 mL (IQR, 2,781-3,253), resulting in a median net UF of 2,498 mL (IQR, 2,249-2,768). The median serum sodium concentration was 131.0 mmol/L (IQR, 127.5-133.7). The median total quantity of sodium removed at the end of the 24-hour dwell time was 387 mmol (IQR, 372-434) (Fig 3). With 387 mmol of sodium removed and 2,498 mL of water removed, the effective sodium concentration of the ultrafiltrate was significantly hypertonic to plasma (Fig 3).

## Discussion

The key findings from experiments with a novel zero-sodium icodextrin and dextrose IP solution in 4 different animal models and in humans are as follows: (1) ascending concentrations of icodextrin and dextrose significantly increased UF; (2) a 30% icodextrin and 10% dextrose solution produced more than 3-fold greater sodium removal and UF volume compared with commercially available PD solutions; (3) chronic exposure of animals to a 30% icodextrin and 10% dextrose IP solution did not result in peritoneal tissue changes compared with control PD solutions; and (4) in humans, a 30% icodextrin and 10% dextrose IP solution was safe and removed 387 mmol (∼9 g) of sodium over 24 hours. Collectively, these foundational results support future studies of a 30% icodextrin and 10% dextrose IP solution as a nonrenal sodium removal therapy for patients with edematous disorders such as HF.

Icodextrin is Food and Drug Administration approved for peritoneal administration at lower concentrations to prevent postsurgical adhesions (ADEPT [4% solution]; Baxter Healthcare Corporation) and as a PD solution (EXTRANEAL [7.5% solution]; Baxter Healthcare Corporation).^1^^,^^10^ However, these solutions contain nearly isotonic sodium concentrations (132-133 mmol/L).^1^^,^^10^ Given the established safety of a 7.5% icodextrin PD solution, a 30% icodextrin and 10% dextrose solution was formulated. One concern of higher icodextrin and dextrose concentrations is the effect on peritoneal health. The IP glucose concentration in humans was only greater than standard PD solutions for a brief period, and the IP icodextrin concentrations were comparable to 7.5% icodextrin PD therapy for the majority of the 24-hour dwell time.^11^ Chronic exposure to these higher concentrations was not associated with peritoneal abnormalities in animal models.

The 30% icodextrin and 10% dextrose IP solution optimized sodium and fluid removal. Dextrose produces UF via aquaporin-1 and small pores, whereas icodextrin generates fluid transport via small pores, minimizing back filtration and sodium sieving after dextrose is metabolized or systemically absorbed.^5^ Icodextrin increases the net UF volume and sodium removal compared with dextrose peritoneal solutions, and combinations of icodextrin with dextrose synergistically increase sodium and fluid removal.^5^^,^^6^^,^^12^ In our previous experiments in humans, 1 L of a 10% dextrose, zero-sodium peritoneal solution for 2 hours removed on average 4.5 g of sodium with a net UF volume of 700 mL, producing a hypertonic net ultrafiltrate sodium concentration of 280 mmol/L.^7^ By removing substantially more sodium than water, zero-sodium dextrose–based solutions rely on the kidneys to eliminate free water and can potentially cause hyponatremia with repeated administration. In contrast, the 30% icodextrin and 10% dextrose solution removed ∼9 g of sodium with a net UF volume of ∼2,500 mL, producing a mildly hypertonic net ultrafiltrate sodium concentration of ∼160 mmol/L. Furthermore, the 10% dextrose IP solution produced rapid UF, with most sodium removal occurring in the first 2 hours of a 6-hour dwell time.^7^ In agreement with previous literature,^5^ we found that a 30% icodextrin and 10% dextrose solution resulted in a more controlled and sustained UF rate over 24 hours, potentially allowing a single dwell with sustained efficacy for a longer duration and intermittent treatments. Finally, the enhanced efficiency of a 30% icodextrin and 10% dextrose solution may facilitate smaller peritoneal infusion volumes. In this study, a 500-mL icodextrin and dextrose solution produced greater sodium and fluid removal than a 1,000-mL dextrose solution in our previous animal models.^7^

Our findings have application to the treatment of humans with edematous disorders such as HF. Excess sodium and water are the predominant cause of HF symptoms and hospitalization, and loop diuretics are the cornerstone therapy for sodium and water removal.^13^, ^14^, ^15^ Loop diuretic resistance is common, causing highly variable diuretic-induced natriuresis.^16^, ^17^, ^18^, ^19^, ^20^ In the Renal Optimization Strategies Evaluation in Acute Heart Failure trial, high-dose loop diuretics produced a median 24-hour sodium output of 3.6 g (IQR, 1.9-6.0).^17^^,^^21^ By comparison, a 2-hour dwell time of a 10% dextrose peritoneal solution removed >4 g of sodium and a 24-hour dwell time of a 30% icodextrin and 10% dextrose solution removed ∼9 g of sodium.^7^ Importantly, 29% of the Renal Optimization Strategies Evaluation in Acute Heart Failure trial population treated with diuretics had a positive sodium balance, which was associated with increased mortality.^17^ Given the large quantity of sodium removed and absence of significant intersubject variability, peritoneal sodium removal may have a substantial advantage over diuretics in patients with HF and diuretic resistance. Likewise, patients receiving traditional PD complicated by refractory hypervolemia may benefit from intermittent therapy.

Several limitations exist. These animal and human experiments were conducted to provide a proof of concept for higher icodextrin peritoneal solutions for sodium removal, not to establish a new indication for the current icodextrin peritoneal solutions in humans. Other animal species, such as mice, may have significantly more icodextrin metabolism in the peritoneum than humans. The inhuman experiments were with a single dose in patients with kidney failure. The long-term safety with chronic therapy and the efficacy in patients without kidney failure were not established and require future studies. Although patients were provided a 2-g/d sodium-restricted diet during the study visit, variations in dietary sodium intake before and during the study visit could influence sodium removal.

In conclusion, a novel, zero-sodium, 30% icodextrin and 10% dextrose IP solution provides significantly greater UF and sodium removal than traditional dialysate solutions. The promising in human safety and efficacy results warrant future investigation as sodium removal therapy in patients with HF.

## Supplementary Materials

Supplementary File (PDF) Figures S1-S6; Items S1-S5.
