# Supplementary material for: Development of a Novel Intraperitoneal Icodextrin/Dextrose Solution for Enhanced Sodium Removal
Source: Kidney Med. 2024 Nov 16;7(1):100938. doi: 10.1016/j.xkme.2024.100938 (PMC11714391; doi:10.1016/j.xkme.2024.100938)
Supplement: Supplementary File (PDF) — Figures S1-S6; Items S1-S5. [file mmc1.pdf]

Item S1: Methods for animal experiments

Experiment in rats

Naïve Sprague Dawley rats (*Rattus norvegicus*) (N=114) were injected intraperitoneally with 10 ml of water-based test solution containing combinations of icodextrin (7.5%, 15%, 25%, 30%) and dextrose (no dextrose, 5% or 10%). Treatment groups and sizes were: 17 receiving 7.5% icodextrin, 21 receiving 15% icodextrin, 16 receiving 20% icodextrin, 19 receiving 25% icodextrin, and 17 receiving 30% icodextrin. Each icodextrin group was divided in 3 subgroups with no, 5%, or 10% dextrose. The control group included 24 rats receiving 10% dextrose. Dwell time was 5 hours based on preliminary experiments showing similar UF volume at 4 and 6 hours, implying peak at 5 hours. At the end of the 5-hour dwell, animals were euthanized and weighed. The abdomen was then opened, fluid drained completely by gravity/manual expression followed by swabbing of the cavity with dry gauze, and the animals were weighed again. The difference between these two weights represented the intraperitoneal volume (instilled fluid + UF). Rats with fluid-filled ceca or diarrhea, indicating intra-cecal injection, were excluded from the analysis (explaining the differing sizes of groups).

Experiment in sheep and pigs

The primary outcome was fluid volume and sodium removal kinetics. Six naïve Dorset sheep (*Ovis aries*) were anesthetized with a combination of intramuscular ketamine, xylazine, and tiletamine/zolazepam (Telazol), intubated, and maintained on inhaled isoflurane. Animals (n=3 in each group) were exposed to two different IP solutions: 30% icodextrin/10% dextrose or 7.5% standard icodextrin infusate (Extraneal, Baxter Healthcare LTD). Two standard PD catheters were placed in the hepatic gutter and pelvis via a small laparotomy. A 500 mL IP solution infusate was instilled into the abdomen with 5 -10 microcuries of I-131 albumin (Daxor Inc., New York, NY) added as an indicator for dilution to determine the kinetics of sodium and

fluid removal over time. IP fluid was sampled (1mL) in duplicate every 15 min until 2 hours, then every 30 min until 180 minutes, then hourly until 8 hours. The ~~pigs~~ were euthanized at the end of 8-hour dwell time, fluid drained via suction, and the total fluid volume was measured.

Six naïve Yorkshire (farm) pigs (*Sus scrofa*) were anesthetized with a combination of intramuscular ketamine, xylazine, and tiletamine/zolazepam (Telazol), intubated, and maintained on inhaled isoflurane. The experimental setup (3 animals randomized to 30% icodextrin/10% dextrose or 7.5% icodextrin standard infusate; dwell time 8 hours), procedures, infusate volume, and sampling were identical to the sheep experiment described above.

GLP Experiments on chronic intraperitoneal health

To evaluate the effects of chronic exposure to 30% icodextrin and 10% dextrose IP solution on the peritoneum, kidneys, omentum, and peritoneal cavity, experiments in mice and sheep were conducted. Mice experiments were conducted at NAMSA (Minneapolis, MN), a facility compliant with U.S. FDA Good Laboratory practices (Regulations, 21 CFR Part 58). Mice (Breed C57BL/6) (N=42) were randomly divided into 2 treatment groups (N=21 per group with equal sex distribution) to receive 30% icodextrin and 10% dextrose IP solution or 4.25% Dextrose Dianeal Low Calcium (2.5 mEq/L) PD Solution (control group). Treatment groups were administered repeated daily doses of the IP solution (Subgroup 1: 2mL; Subgroup 2: 1mL, Subgroup 3: 0.5mL) via IP catheter over 30 days. The animals were observed for signs of toxicity immediately after injection and daily throughout the study duration. Body weights were measured prior to the initial administration, weekly, and prior to termination. At the end of the 30-day survival period, blood was collected for hematology and serum chemistry and the animals were humanely euthanized. A limited necropsy was performed, including the examination of the kidneys, peritoneum, omentum, peritoneal cavity, and abdominal organs. Select tissues were

collected for histopathological analysis. Overall interpretation was based on the incidence and severity of abnormalities including behavioral and clinical abnormalities, body weight changes, mortality, and gross observations during necropsy, clinical pathology, and histopathological evaluation as compared to control mice.

Sheep experiments were conducted at WuXi AppTec (Suzhou) in Jiangsu, China, a facility compliant with U.S. FDA Good Laboratory practices (Regulations, 21 CFR Part 58). Small-tailed Han sheep (*Ovis aries*) (N=18) with weights ranging 37.0 – 64.8kg were randomly divided into 3 treatment groups (N=6 per group) to receive 30% icodextrin and 10% dextrose IP solution 10mL/kg (Group 1), 20mL/kg (Group 2), or 4.25% Dextrose Dianeal Low Calcium (2.5 mEq/L) PD Solution 40mL/kg (Group 3 or "control group"). Sheep had two catheters placed in the peritoneal space for serial IP solution administration and an intravenous catheter placed for fluid or electrolyte repletion as needed. Groups 1 and 3 (control group) were treated daily for 45 days. Due to animal welfare concerns because of massive fluid removal and repletion, Group 2 was treated for 30 days. Every 24 hours, the peritoneal cavity was emptied of fluid prior to solution administration via PD catheters. Complete blood counts and serum chemistries were measured at baseline, mid-treatment period, and at time of necropsy. At the end of the treatment period, animals were euthanized, and necropsies were performed. Gross lesions were recorded, and tissue samples were collected from the kidney, peritoneum, and omentum. Tissue samples were embedded in paraffin blocks, sectioned at 4-6 μm thickness, deposited on slides, and stained with hematoxylin and eosin (H&E). Slides were evaluated by a veterinary pathologist for the presence of fibrosis, angiogenesis, or any findings abnormal for the respective type of host tissue.

Item S2: Methods for phase 1 study in humans

Inclusion criteria

Patients meeting the following criteria were eligible for participation in the study:

- Patient's treatment covered by the Mexican Social Security Department.
- Adults over the age of eighteen.
- Patients with chronic kidney failure under peritoneal dialysis treatment.
- Patients with a functional peritoneal dialysis catheter.
- Patient's peritoneal dialysis prescription not changed in the previous month (ongoing peritoneal dialysis as an outpatient or ongoing automated peritoneal dialysis).
- Patients with stated wish to participate
- Signed informed consent.
- Peritoneal dialysis catheter and the transfer line compatible with the peritoneal dialysis bag (Luer male connector).
- Patient clinically euvolemic as assessed by treating physician.
- No change to peritoneal dialysis prescription since performance of a peritoneal equilibrium test.

Exclusion Criteria

Patients meeting any of the following criteria were considered ineligible:

- Diabetes mellitus type 1 or uncontrolled diabetes type 2 (Hemoglobin A1c > 8%)
- Active infection.
- Serum sodium prior to the study <130 mmol/l.
- Serum bicarbonate prior to the study <18 mmol/l.
- PD prescription using only solutions with 1.5% dextrose (lowest concentration available).
- Hemoglobin <8 g/dl
- Active bleeding.
- PD prescription using a 4.25% dextrose solution at least once a day.
- Membrane defect or mechanical defect.
- Diastolic dysfunction with an increase in filling pressure according to the echocardiogram. (E/A ratio of > 2.5 or an E/E ratio of > 15).
- Active or suspected peritonitis as assessed by treating physician.

Laboratory measurements

Blood samples taken at baseline and at the end of the 24-hour dwell time were analyzed for urea, creatinine, glucose, uric acid, magnesium, calcium, phosphorus, bicarbonate and albumin, globulin, total protein, total bilirubin, alkaline phosphatase, lactic dehydrogenase at the central

laboratory of the IMSS Centro Medico Nacional (CMN) Siglo XXI Cardiology Hospital, Mexico City, Mexico.

Blood samples taken during dwell time were analyzed immediately using a point-of-care analysis device (Instrumentation Laboratory Gem Premier 403500 Blood Gas Analyzer) for pH, pO2, PCO2, bicarbonate, sodium, potassium, and hemoglobin.

Primary peritoneal liquid samples labelled with subject ID and timepoint of collection were collected and shipped to Yale University for analysis of electrolytes, icodextrin, and other analytes. Plasma samples were shipped to Yale University for analysis of icodextrin. Electrolytes and glucose concentrations in PD fluid were determined using a Roche fully automated chemistry autoanalyzer (Roche Diagnostics, Indianapolis, USA). Peritoneal volumes were calculated based on icodextrin concentration, the known instilled volume, and total ultrafiltration volume. Icodextrin in PD fluid and ~~serum~~ was measured following hydrolysis of all glucose polymers to glucose using amyloglucosidase. Briefly, 100 µl of infusate was incubated with 500 µl of ~~amyloglucodisade~~ (6 mg/ml) at 55ºC for 30 minutes, the samples were diluted 20-fold for infusate and 5-fold for plasma. Glucose was measured using the Roche analyzer.

The treatment was to be considered safe if a subject did not experience any serious adverse events related to the infusate (defined according to Official Mexican Standard 012-SSA3-2012).

Figure S1: Phase 1 study in humans

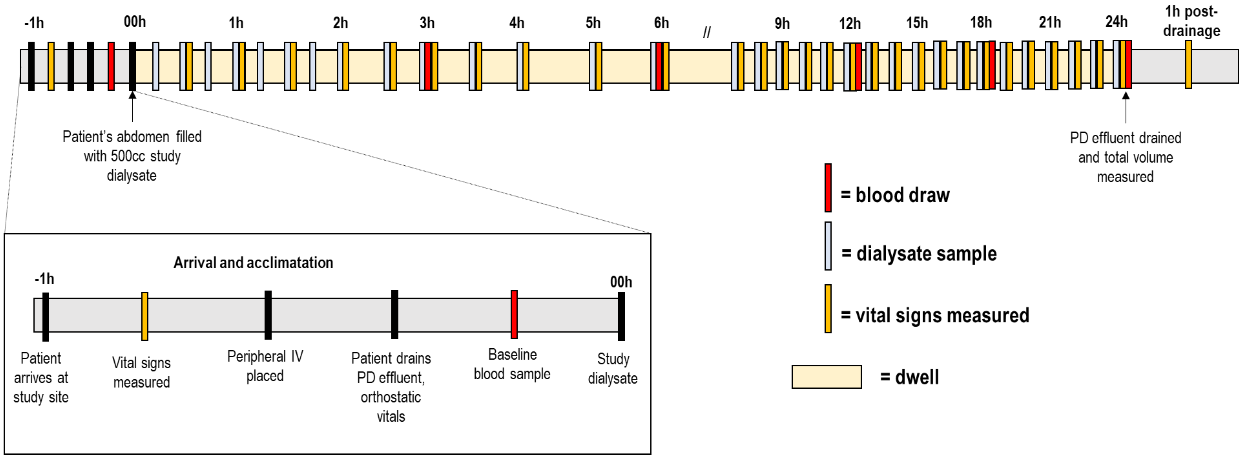

Figure S1 Legend: Study diagram shows the procedures across the 24-hour dwell time.

Figure S2: Ultrafiltration across various combinations of dextrose and icodextrin combinations in rats

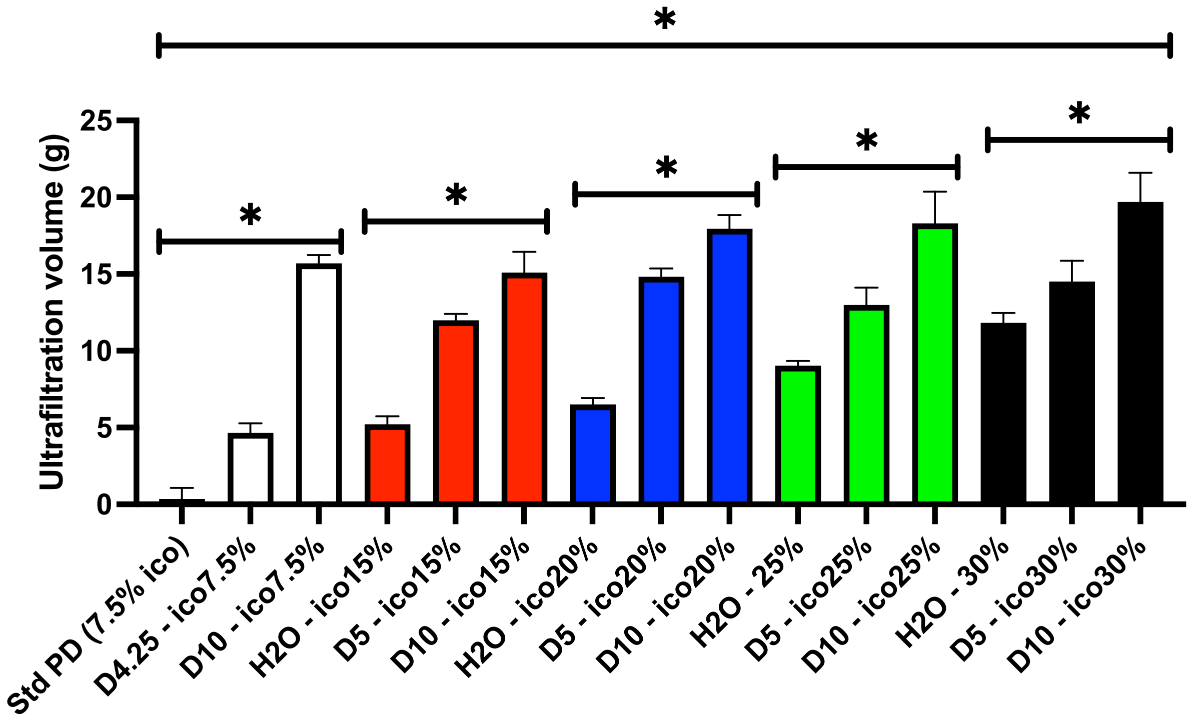

Figure S2 Legend: In rats, the ultrafiltration volume after 5 hours of dwell time with 10 ml infusate containing different concentrations and combinations of dextrose (D) and icodextrin (ico) is presented. Fluid was measured gravimetrically, and the instilled volume was assumed to be 10g based on the 10ml fluid injection. Bars and error bars represent mean and SEM, respectively. Asterisks indicate statistical significance at $P<0.01$. Std PD = standard peritoneal dialysis solution containing 7.5% icodextrin; H2O= water

Item S3: Results: sheep

Large animal evaluation (sheep)

The UF kinetics with 30% icodextrin/10% dextrose solution were consistent with a rapid early UF from the osmotic load of 10% dextrose, with a slow continued UF over the full 8-hour dwell from the 30% icodextrin. Although UF was relatively minor in the 7.5% icodextrin dialysate solution from hours 4-8, significant UF continued from 4-8 hours with the 30% icodextrin/10% dextrose solution. (Supplemental Figure 3, top left) Total sodium removed was approximately 4 times greater with 30% icodextrin/10% dextrose compared with standard 7.5% icodextrin dialysis solution (7.07±0.72 g vs. 1.78±1.27 g; p=0.003). (Supplemental Figure 3, top right) Peritoneal fluid glucose concentrations over the duration of the dwell were low and as expected with both solutions, in line with a lack of significant intraperitoneal metabolism of icodextrin to glucose in sheep (Supplemental Figure 3, bottom panels).

Figure S3. Kinetics of UF, sodium removal, icodextrin metabolism, and total glucose in sheep peritoneal dialysis fluid

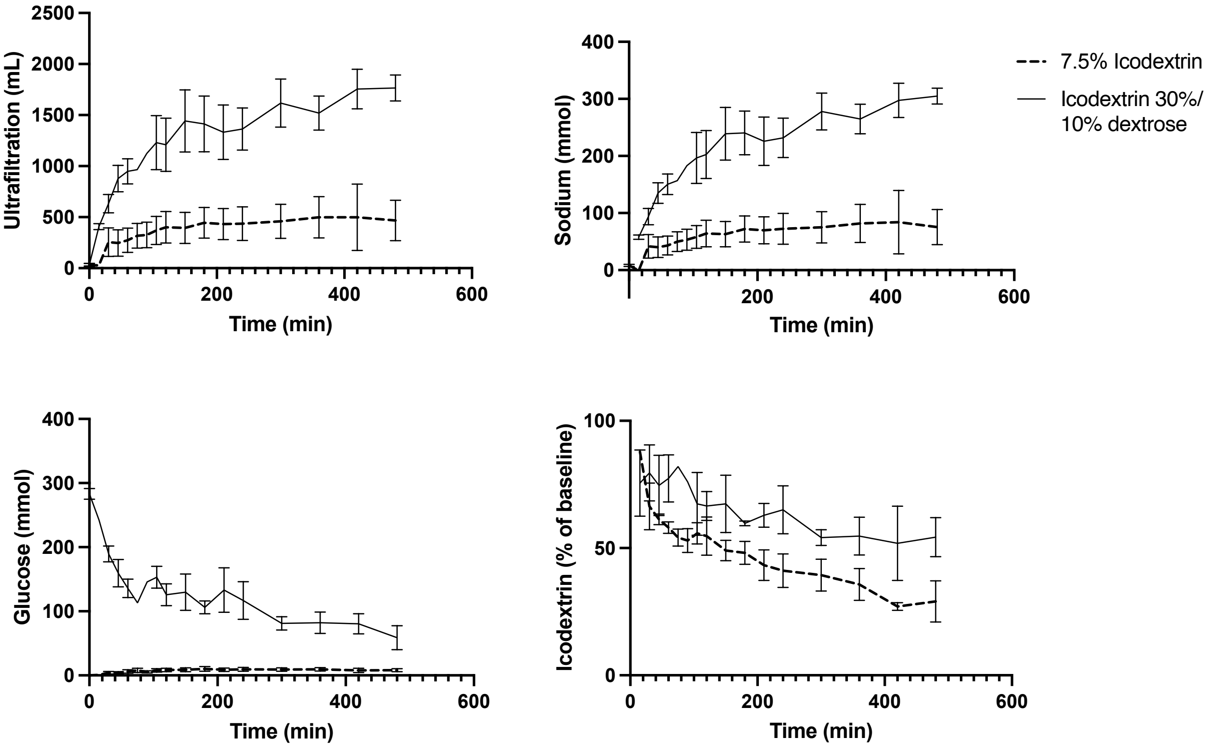

Figure S3 Legend: Sheep that received 30% icodextrin/10% dextrose peritoneal solution compared to a standard 7.5% icodextrin peritoneal dialysis (PD) solution had greater ultrafiltration (top left panel) and total sodium excretion (top right panel). Bottom left panel shows total glucose in ovine peritoneal fluid; as determined by the experimental setup, glucose concentration was higher in the 30% icodextrin/10% dextrose group but decreased over time. Bottom right panel shows total icodextrin in peritoneal fluid as a percentage of baseline icodextrin. As determined by the experimental setup, icodextrin concentration was higher in animals that had received 30% icodextrin/10% dextrose than in those that had received standard 7.5% icodextrin PD solution. Concentration decreased in both groups over time. Data points represent the mean value at each time point, error bars indicate SEM.

Item S4: Results: Pigs

Large animal evaluation (pigs)

Given that intraperitoneal icodextrin metabolism is known to differ between rodents and humans, we next sought evaluation of the 30% icodextrin/10% dextrose combined solution in a large animal model. In pigs treated with 30% icodextrin/10% dextrose infusion, total UF volume was 3 times greater (Mean 2.0±0.21 L vs 0.67±0.25 L; p=0.002) and total sodium removal was 3.5 time greater (7.13±0.81g vs 1.89±0.74 g; p=0.001) compared to pigs infused with 7.5% commercially available icodextrin dialysate solution (**Supplemental Figure 4**). However, there was significant intra-peritoneal icodextrin metabolism in the pigs as peritoneal fluid glucose concentration reached well over 80 mmol/L (1440 mg/dL) in animals treated with commercially available 7.5% icodextrin PD fluid. (**Supplementary Figure 5**). This indicated that swine are not an ideal model to study icodextrin kinetics.

4. Kinetics of ultrafiltration and sodium removal in pigs

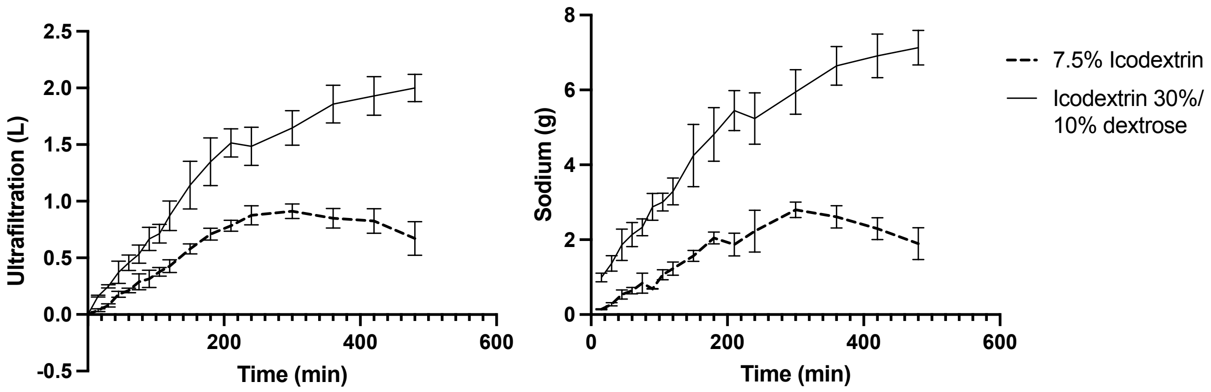

Figure S4: Legend: Left panel shows ultrafiltration as determined by dilution of I-131 albumin added at infusion. Ultrafiltration was higher with 30% icodextrin/10% dextrose in water peritoneal solution compared to 7.5% standard icodextrin peritoneal dialysis (PD) solution. Right panel shows that total sodium excretion across the peritoneal membrane was also higher with 30% icodextrin/10% dextrose in water peritoneal solution. Data points represent mean± SEM.

Figure S5: Glucose concentration in porcine peritoneal fluid

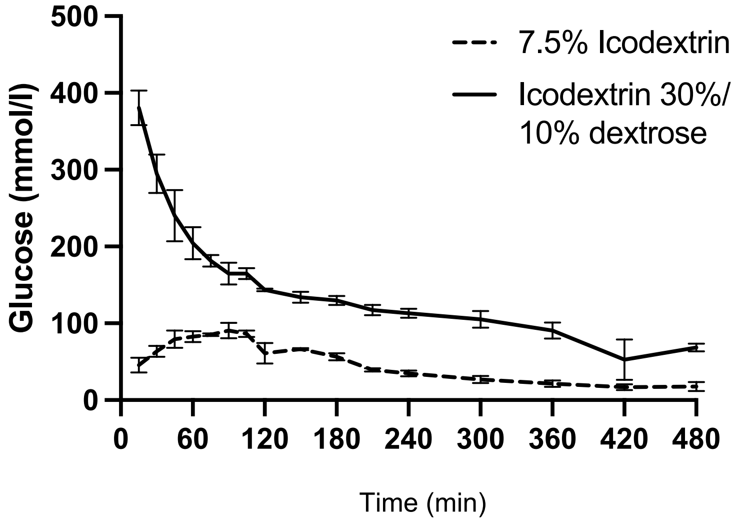

Figure S5 Legend: Glucose concentration in porcine peritoneal fluid. Glucose concentrations in peritoneal fluid were significantly increased in pigs that received 30% icodextrin/10% dextrose infusate compared to those that received a standard 7.5% icodextrin peritoneal dialysis (PD) solution.

Item S5: Results: chronic peritoneal effects of icodextrin

Mouse GLP experiments:

In a total of 42 mice, no systemic toxic effects of long-term 30% icodextrin/10% dextrose IP solution were observed compared to a standard 4.25% dextrose PD solution. Body weights were comparable between treatment groups (P>0.05). No abnormalities in hematology or serum chemistries were observed between treatment groups (P> 0.05 for all). At necropsy, no abnormalities in gross pathology were observed for any mice. Histopathology of the kidneys, peritoneum, and omentum did not show any significant changes nor differences between treatment groups.

Sheep GLP experiments:

In a total of 18 sheep, no systemic toxic effects at 45 days of 10mL/kg (n=5 completing) and 30 days of 20mL/kg (n=6 completing) 30% icodextrin/10% dextrose IP solution were observed compared to 45 days of a standard (control) 4.25% dextrose PD solution (n=5 completing). In Group 1, one sheep was euthanized early (Treatment Day 33) and underwent necropsy. In Group 2, 6 completed the study. Although one sheep died unexpectedly (Treatment Day 6), this sheep was replaced with another sheep so that 6 sheep completed the study. In Group 3 (control), one sheep was euthanized early (Treatment Day 25) and underwent necropsy. No abnormalities in hematology or serum chemistries were observed between treatment groups (P> 0.05 for all). No significant differences in the gross appearance of peritoneum, kidney, omentum or peritoneal cavity were observed between groups. Histopathology of the kidneys, peritoneum, and omentum did not differ between treatment groups.

Figure S6. Perceived pain scores over the 24-hour dwell in humans

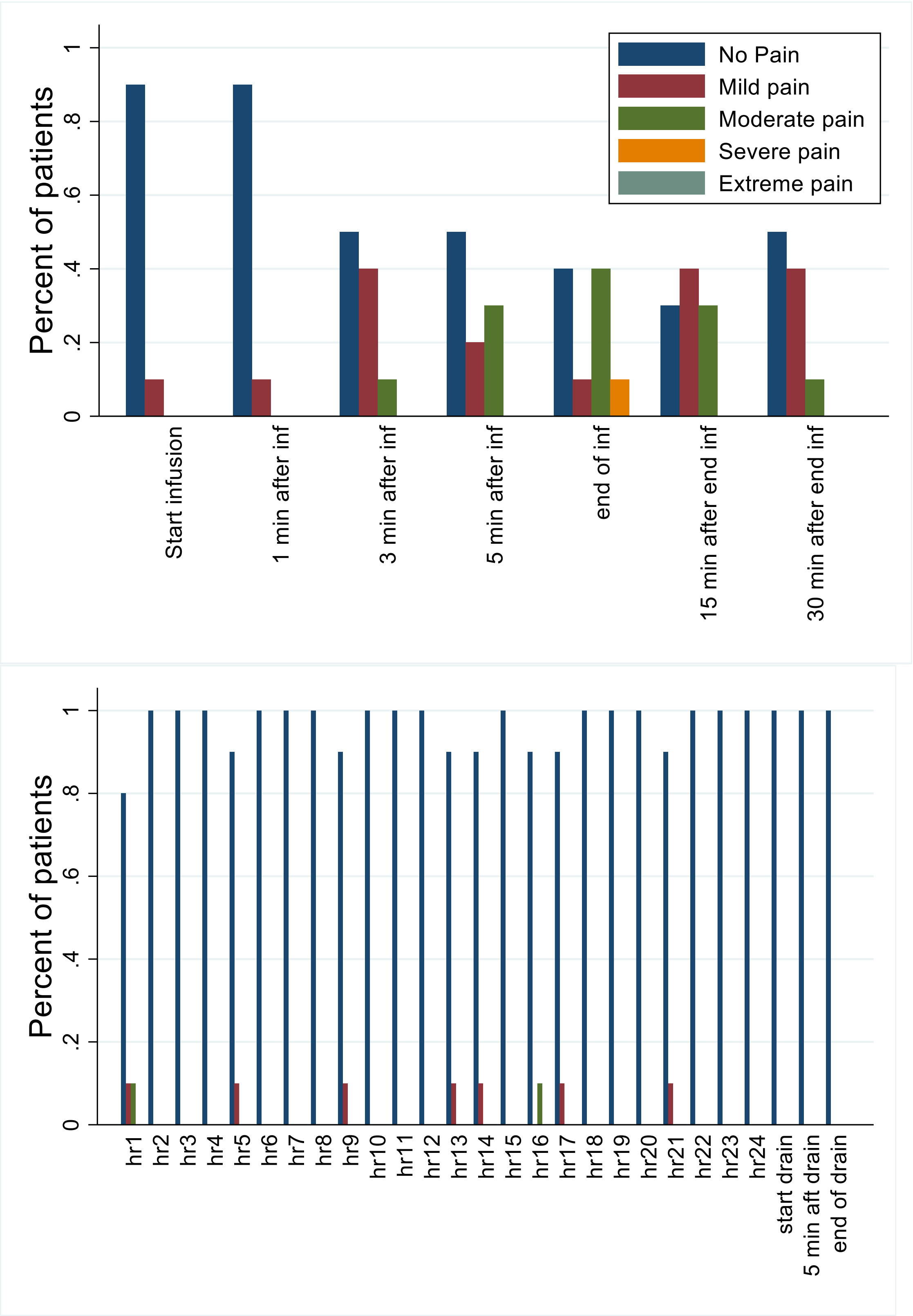

Figure S6 Legend: Top panel shows the percentage of patients reporting pain from the start of infusion to 30 minutes after end of the intraperitoneal infusion. Bottom panel shows the percentage of patients reporting pain from hour-1 to end of intraperitoneal fluid drain over the 24-hour dwell time. Blue = no pain; Red = mild pain (1 out of 4 on pain scale); Green = moderate pain (2 out of 4 on pain scale); Orange = severe pain (3 out of 4 on pain scale); Gray = extreme pain (4 out of 4 on pain scale).
